# Modeling the Effect of Hypoxia and DNA Repair Inhibition on Cell Survival after Photon Irradiation

**DOI:** 10.3390/ijms20236054

**Published:** 2019-11-30

**Authors:** Hans Liew, Carmen Klein, Frank T. Zenke, Amir Abdollahi, Jürgen Debus, Ivana Dokic, Andrea Mairani

**Affiliations:** 1 Clinical Cooperation Unit Radiation Oncology, German Cancer Research Center (DKFZ), 69120 Heidelberg, Germany; h.liew@dkfz-heidelberg.de (H.L.); Juergen.Debus@med.uni-heidelberg.de (J.D.); 2 Division of Molecular and Translational Radiation Oncology, National Center for Tumor Diseases (NCT), Heidelberg University Hospital, 69120 Heidelberg, Germany; carmen.klein@dkfz-heidelberg.de (C.K.); a.amir@dkfz-heidelberg.de (A.A.); 3 Heidelberg Institute of Radiation Oncology (HIRO), German Cancer Research Center (DKFZ), 69120 Heidelberg, Germany; 4 German Cancer Consortium (DKTK), 69120 Heidelberg, Germany; 5 Heidelberg Ion-Beam Therapy Center (HIT), 69120 Heidelberg, Germany; 6 Faculty of Physics and Astronomy, Heidelberg University, 69120 Heidelberg, Germany; 7 Merck KGaA, 64293 Darmstadt, Germany; Frank.Zenke@merckgroup.com

**Keywords:** ionizing radiation, DNA repair, hypoxia, modeling, radiosensitizer

## Abstract

Mechanistic approaches to modeling the effects of ionizing radiation on cells are on the rise, promising a better understanding of predictions and higher flexibility concerning conditions to be accounted for. In this work we modified and extended a previously published mechanistic model of cell survival after photon irradiation under hypoxia to account for radiosensitization caused by deficiency or inhibition of DNA damage repair enzymes. The model is shown to be capable of describing the survival data of cells with DNA damage repair deficiency, both under norm- and hypoxia. We find that our parameterization of radiosensitization is invariant under change of oxygen status, indicating that the relevant parameters for both mechanisms can be obtained independently and introduced freely to the model to predict their combined effect.

## 1. Introduction

Radiation therapy is one of the cornerstones of cancer care, where ~50% of patents receive radiation during the course of disease [1]. Radiobiological modeling is an integral part of radiation oncology and radiation therapy, used to predict normal and tumor tissue response, which is of particular significance when moving towards personalized radiation treatment, including treatment gap corrections, normal tissue tolerance predictions, optimization of therapy determined by predictive assays, multi-modality schedule design, and the simulation of clinical trials [2]. In this work, we present the first step towards development of our modeling platform called “UNIfied and VERSatile Engine” (UNIVERSE), within which we aim to integrate multiple biological responses and mechanisms relevant for describing radiation response of different cell types. In this manuscript, we focus on describing the cellular response of a particularly radioresistant tumor sub-population—hypoxic cells. Cells under hypoxic conditions exhibit increased radioresistance, and tumors containing hypoxic regions have significantly worse chances of successful treatment with radiotherapy [3,4,5]. The increased radiosensitivity of cells in the presence of free oxygen is usually explained by the oxygen fixation hypothesis: Molecular oxygen has the ability to react with radicals produced in the DNA, thereby fixating the damage, preventing the direct chemical restoration of the DNA radical by reacting with H⁺ [3]. The concept of the oxygen enhancement ratio (OER) is classically used to quantify the dependence of cell survival on the oxygenation status. The OER is usually defined as the ratio between the doses needed to induce the same survival fraction in a hypoxic and a normoxic environment [6]. One of the key strategies to overcome the radioresistance in hypoxic cells is a dual treatment, i.e., a combined treatment with photon irradiation and administration of radiosensitizing drugs, such as DNA damage response (DDR) inhibitors [7,8,9,10].

To model the response of hypoxic tumor cells to dual treatment, we based our approach on a previously published model of our group [11]. Similarly to models described by other groups [12,13,14], the here presented model divides the cells’ nucleus into equally sized subvolumes containing about 2 Mbp, dubbed giant loops [15,16,17]. Those giant loops containing either exactly one or two and more double strand breaks (DSB) are classified as isolated DSB (iDSB) or complex DSB (cDSB), respectively. Giant loops have been identified as possible critical targets, inside which multiple lesions resist swift repair [18,19,20,21]. It was shown that computed numbers of iDSB ($N_{iDSB}$) and cDSB ($N_{cDSB}$) matched well with observed frequencies of quickly (iDSB) and slowly (cDSB) repaired DSB in rejoining studies [22,23]. The core model of our choice [11,13] associates both classes of lesions with so-called lethality parameters, $K_{iDSB}$ and $K_{cDSB}$, respectively. These parameters are the corresponding probabilities for lesions of a given class to become lethal, meaning the cell loses its potential to further proliferate. Complex lesions are considered to be significantly more lethal than isolated lesions, as each of them poses a high risk for chromatin loss [14] and they remain unrepaired for a prolonged time [24,25,26]. It is indeed found that $K_{cDSB}$ is several magnitudes larger than $K_{iDSB}$ in cases derived from experimental data [11,26].

Regarding the hypoxic cell population, Carlson et al. [6] made compelling arguments for an interpretation of the OER as the ratio of doses needed to induce the same total amount of DSBs in hypoxic and normoxic cells. They further suggested the replacement of the OER term with the name hypoxia reduction factor ($HRF_{DSB}^{O_{2}}$) at a given oxygen concentration $\left[O_{2}\right]$ within this context [27]. In line with the above ideas, we introduced the $HRF_{DSB}^{O_{2}}$ as a parameter into our model, which solely modifies the initial total yield of DSB ($N_{tDSB}$). Following evidence that the oxygen status has no effect on the DSB rejoining rates [6], the lethality parameters were assumed to be invariant under the change of oxygenation. We could show in our previous work, that the sole introduction of the $HRF_{DSB}^{O_{2}}$ parameter was sufficient to describe survival data from literature and that the derived $HRF_{DSB}^{O_{2}}$ values were well described by a parameterization suggested by Carlson et al. [6] (a function of oxygen concentration $\left[O_{2}\right]$, inspired by the initial studies of Alper and Howard-Flanders [28]) [11]. In the work presented here, we investigate whether our model is capable of describing an experimental set of cell survival data containing five cell lines at three different oxygen levels each, and whether the derived $HRF_{DSB}^{O_{2}}$ values are in accordance with the formerly introduced parameterization.

To further extend the model and describe the particular case of hypoxic tumor cells response to dual treatment, radiotherapy, and DDR inhibition, we introduced a so-called radiosensitization factor (*RSF*) that modifies $K_{iDSB}$. This is based on observations by Hufnagl et al. [26], that the increased radiosensitivity of repair-deficient cell lines could be accounted for by increasing the lethality parameter of isolated DSB, while keeping the lethality parameter for complex DSB constant. Complex DSB are argued to pose such severe challenge to the DDR that any change in the repair capabilities of the cell has no effect on their lethality parameter. To validate this extension, we benchmarked our model in two scenarios, using experimental data obtained from cells in which one of the two key radiation-induced DDR molecules, DNA-dependent protein kinase (DNA-PK) or ataxia-telangiectasia mutated (ATM), was impaired [29]. First, we studied the robustness of the model to predict survival data of DNA-PK-deficient mutants of CHO cells. Secondly, we tested the model to predict the survival of two human lung cancer cell lines with pharmacologically-inhibited ATM. Both scenarios were investigated under normoxia and hypoxia.

## 2. Results

### 2.1. Modeling Hypoxia-Induced Radioresistence

**Figure 1:**
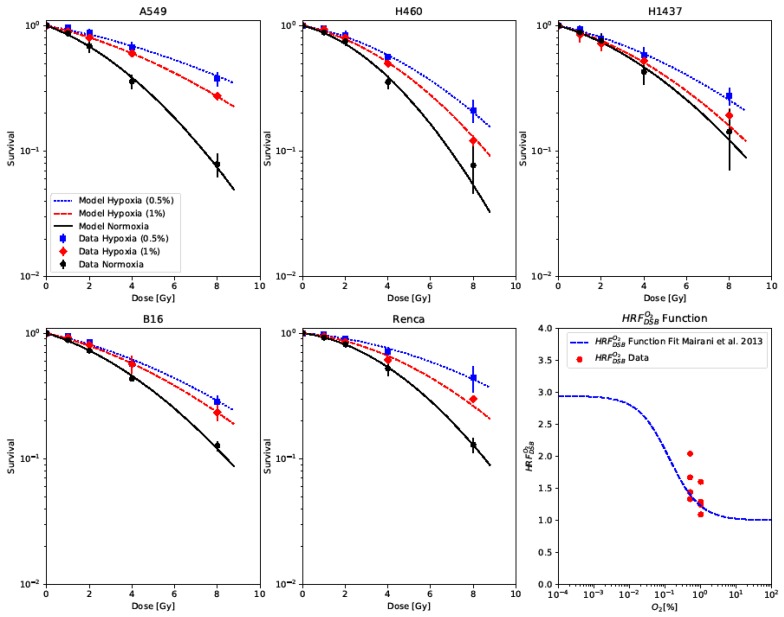
Cell survival data of five cell lines irradiated under normoxia (black) and under two hypoxia levels (0.5% and 1% [$O_{2}$], blue and red) fitted by the model. Lower-right panel: The derived $HRF_{DSB}^{O_{2}}$ values compared to a parametrization introduced in [11].

Our survival data for five different cell lines (A549, H460, H1437, B16, Renca) exposed to three distinct oxygenation levels (normoxia 20% [$O_{2}$], 1% [$O_{2}$], 0.5% [$O_{2}$]) were fitted using our model (Figure 1). $K_{iDSB}$ and $K_{cDSB}$ were fitted for each cell line as free parameters to the normoxic data. Keeping these parameters fixed, for each cell line, the specific $HRF_{DSB}^{O_{2}}$ for the two hypoxic conditions were fitted. The determined numerical values of each parameter can be found in Table 1. Our model shows excellent capability in describing the acquired data, indicating consistency with our earlier work [11]. Furthermore, the $HRF_{DSB}^{O_{2}}$ values derived from our data were in accordance with the parametrization of $HRF_{DSB}^{O_{2}}$ as a function of oxygen concentration published in [11], as shown in the bottom right panel of Figure 1.

### 2.2. Modeling Cell Survival of DNA-PK-Impaired Cell Lines

**Figure 2:**
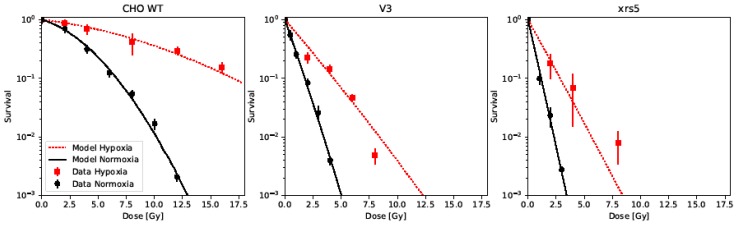
Cell survival data of CHO WT cells and two DNA-PKcs response-deficient mutants (V3 and xrs5) irradiated under normoxia (black) and hypoxia (<1% [$O_{2}$], red), taken from [30] predicted by the model.

Survival data of the CHO cell line and two of its DNA-PK response-deficient mutants (V3 cell line is DNA-PKcs-deficient and xrs-5 cell line is Ku80-deficient) under normoxia and hypoxia were gathered from Cartwright et al. (Figure 2) [30]. The cell line-specific parameters $K_{iDSB}$ and $K_{cDSB}$ were found by fitting our model to the normoxic survival data of the wild-type cells. The $HRF_{DSB}^{O_{2}}$ of the cell line was then derived by fitting the model to the hypoxic survival data, keeping the aforementioned lethality parameters ($K_{iDSB}$ and $K_{cDSB}$) fixed. By increasing only the lethality parameter of isolated DSB ($K_{iDSB}$) by a radiosensitization factor (*RSF*) for each of the mutant cell lines, their survival under normoxia was able to be fitted accurately. The numerical values of the parameters can be found in Table 2. By applying the $HRF_{DSB}^{O_{2}}$ derived from the wild-type data and the *RSF* derived for each mutant under normoxia, the survival of the two mutant cell lines under hypoxia could be predicted satisfactorily. However, deviations can be observed at the highest reported doses.

### 2.3. Modeling Cell Survival of Cell Lines with Pharmacologically-Inhibited ATM

**Figure 3:**
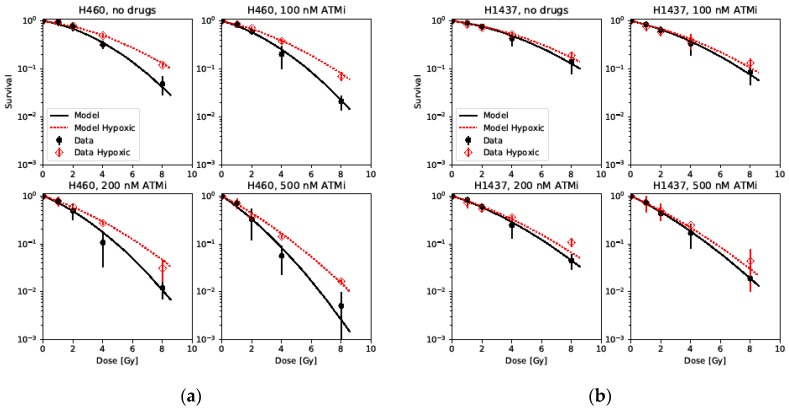
Cell survival data of two cell lines, (**a**) H460 and (**b**) H1437, irradiated under normoxia (black) and hypoxia (1% [$O_{2}$], red) after exposure to different concentrations of an ataxia-telangiectasia mutated inhibitor (ATMi) fitted by the model.

The same approach was applied to our data containing two of the initially presented cell lines (H460 and H1437) exposed to different concentrations of an ATM inhibitor (ATMi) and irradiated under normoxia and hypoxia (Figure 3). First, the cell line-specific lethality parameters ($K_{iDSB}$ and $K_{cDSB}$) were derived by fitting our model to the data of cells irradiated under normoxia and without drug treatment. Second, the $HRF_{DSB}^{O_{2}}$ of each cell line was derived by fitting the model to the data of cells receiving no drug but irradiated under hypoxia, keeping the lethality parameters $K_{iDSB}$ and $K_{cDSB}$ fixed. Third, *RSF*s for the lethality parameters of the isolated lesions ($K_{iDSB}$) were derived for each cell line and drug concentration by fitting the model to the data of cells at each drug concentration under normoxia. The numerical values of the derived parameters can be found in Table 3. Again, by applying the $HRF_{DSB}^{O_{2}}$ derived from the non-treated cells and the *RSF* values found for each drug concentration irradiated under normoxia, the survival of the two cell lines exposed to the combination of different drug concentrations and hypoxia could be predicted very well.

## 3. Discussion

Our model provided an excellent description of the survival data of five cell lines and three oxygen levels presented in Figure 1, confirming the applicability of the previously published framework [11]. It underlines the hypothesis introduced in our former work, that cell survival under hypoxic conditions can be described in a first approximation by keeping the defined lethality of isolated and complex lesions invariant and only modifying the overall induction of DSBs by a given factor ($HRF_{DSB}^{O_{2}}$). In contrast to our former publication, we determined the lethality parameters $K_{iDSB}$ and $K_{cDSB}$ by fitting them to the experimental data. This practice might lead to better predictions, as up to now, these values were recalculated from provided or fitted LQ model parameters (α and β values), based on a Taylor expansion at low doses of our model equations. However such approximate recalculations remain to be crucial in cases in which only the α and β values are available but not the full set of cell survival data. Furthermore, we were able to show that the derived $HRF_{DSB}^{O_{2}}$ values from our data coincided well with the $HRF_{DSB}^{O_{2}}$ parametrization as a function of oxygen concentration introduced in our former publication. The highest $HRF_{DSB}^{O_{2}}$ value for both oxygen levels was obtained from A549 cells. Including these data points, the mean of the derived $HRF_{DSB}^{O_{2}}$ values deviated by 0.08 and 0.19 for 1% $\left[O_{2}\right]$ and 0.5% $\left[O_{2}\right]$, respectively. If one excludes the A549 data, the mean of the derived $HRF_{DSB}^{O_{2}}$ values only deviated from the prediction by 0.01 and 0.07 for 1% $\left[O_{2}\right]$ and 0.5% $\left[O_{2}\right]$, respectively. This is further evidence for this parameterization to be a widely applicable estimate for the $HRF_{DSB}^{O_{2}}$ in cases where the data to derive the exact value are not available.

The idea of increasing the lethality of isolated lesions $K_{iDSB}$ under the presented model in order to describe the increased cell killing observed for repair deficient cell lines has already been expressed and successfully demonstrated by Hufnagl et al. within the GLOBLE model [26]. They consider the lethality of complex lesions $K_{cDSB}$ as being fixed, as each complex lesion poses a “significant burden for the cell”, irrespective of the DNA damage repair capabilities of the cell. Further, they argue, that in NHEJ deficient cell lines, the lethality of isolated lesions increases depending on the cell cycle status of the cells. While we adapt and fully agree with the notion of a fixed lethality of complex damages independent of the repair capabilities of a cell, we had no information on the cell cycle distributions underlying the data taken from Cartwright et al. [30] and found different radiosensitizing factors (*RSF*) for the two DNA-PK response-deficient mutants analyzed. Therefore we decided to introduce the *RSF* as a free parameter in UNIVERSE, fitted to each mutant cell line. The *RSF* values found for the two CHO mutants V3 and xrs5 indicate that the probability of an isolated lesion to become a lethal lesion increases through DNA-PK response deficiency in these cell lines by a factor of about 10 and 15, respectively. Even though both *RSF* factors are related to the functional DNA-PK repair activity, the difference in the *RSF* of both cell lines might be retraced to the fact, that both are deficient of different enzymes taking part in the DNA-PK response: While xrs-5 cells are deficient of the Ku80 DNA-PK subunit [30,31], V3 cells lack the catalytic subunit for DNA-PK [30,32]. It is, however, unclear if and how the *RSF* is coupled to the activity of both proteins. Furthermore, different extents of remaining expression or compensation by other repair proteins might also lead to the observed difference in the *RSF* values of both mutants. One would have to compare several groups of cell lines with the exact same deficiencies to gain a better understanding of the underlying dependencies. More importantly, we could show that the survival of the mutant cell lines under hypoxia were well predicted by our model by combining the $HRF_{DSB}^{O_{2}}$ determined based on the wild-type data and keeping the *RSF* values derived from the normoxic data of each mutant invariant.

Since both DNA-PK and ATM are essential molecules for irradiation-induced DNA damage repair, recruited to the DNA damage sites [33,34,35], we assumed that pharmacological inhibition of ATM should also lead to an increased lethality of isolated damage sites, similarly to the genetic models in which DNA-PK is deficient, with a concentration-dependent effect. Indeed, the observed survival in cells exposed to different concentrations of the ATM inhibitor under normoxia was described with high fidelity by introducing an *RSF* for isolated lesions. Further, the values of the derived *RSF* illustrate the increasing lethality of isolated lesions with increasing drug concentrations, leading to stronger inhibition of the repair, while staying below the value derived for a pathway deficiency. It is also to be noticed that the *RSF* values for both H460 and H1437 cells are fairly similar. However, more cell lines have to be analyzed to investigate whether this can be extended to a general trend and if the presented method is generally applicable to other repair-inhibiting drugs. Nevertheless, we could show that the *RSF* values derived from normoxic data can accurately describe the survival in hypoxic conditions by introducing the $HRF_{DSB}^{O_{2}}$ derived from cells without drug treatment.

Taken together, we could show that impairment of DNA damage repair, both in repair-deficient cell lines and cells treated with a DDR inhibitor, could be accounted for by a manipulation of the lethality of isolated lesions with an *RSF* in our model. Moreover, this modified lethality could stay invariant under change of oxygen supply, while sustaining good predictive capabilities. Thus, the uniqueness of our approach lies in its capability to describe two separate cell response mechanisms in any combination using minimal input parameters, which can be separately derived. Based on this, we believe that our approach has a high potential to implement further cellular mechanisms in order to produce predictions tailored to diverse clinical scenarios.

## 4. Materials and Methods

### 4.1. Experimental Data from Literature

Experimental data used to benchmark the model on survival of genetically DDR-deficient cell lines were taken from [30]. The data used to benchmark the model on survival of NCI-H460 (H460) and NCI-H1437 (H1437) cells in which the DDR was pharmacologically inhibited using an ATM inhibitor under normoxia and hypoxia, as well as the survival data of A549 cells under hypoxia and normoxia, were taken from [10].

### 4.2. Cell Culture, Clonogenic Survival Assay, and Irradiation

For the validation of our model under hypoxic conditions, additional experiments were performed using Renca (murine renal carcinoma; American Type Culture Collection, Manassas, VA, USA) and B16-Blue ISG (murine melanoma; Invitrogen, Thermo Fischer, Waltham, MA, USA) (B16) cells. Both cell lines were grown in RPMI 1640 Medium (Gibco, Thermo Fischer, Waltham, MA, USA) supplemented with 10% fetal bovine serum (FBS)(Merck Millipore, Darmstadt, Germany) at 37 °C and 5% CO_2_ atmosphere. Experiments in hypoxic conditions were performed at 0.5 or 1% O_2_ and 5% CO_2_ using a custom hypoxic chamber (C-chamber; Biospherix, Parish, NY, USA), including an online monitoring controller for O_2_ and CO_2_ concentrations (ProOx and ProCO2 model; Biospherix, Parish, NY, USA). Fifty cells per well in a 96-well format were seeded not more than 16 h before irradiation. Hypoxic irradiation was performed after incubation for 4 h under respective oxygen conditions. Cells were irradiated in the sealed hypoxia chamber with a dose series of photons of 1, 2, 4, or 8 Gy and thereafter incubated under normoxic conditions. The ATM inhibitor was kindly provided by Merck KGaA, and dissolved in DMSO (PAN-Biotech, Aidenbach, Germany) and diluted in RPMI 1640 medium. The inhibitor was added to H460 and H1437 cells at 100, 200, or 500 nM just before incubation under hypoxia or normoxia started. Controls also contained < 0.1% DMSO. Inhibitors were left in the media for 24 h and then replaced with fresh RPMI 1640 medium and the plates were returned to the incubator for colony formation. After 4 days (A549), 5 days (H460, Renca, and B16), or 7 days (H1437), plates were imaged by an online microscopy system at 4x magnification (IncuCyte, Essen Bioscience, Sartorius, Göttingen, Germany). The images were analyzed by the IncuCyte Zoom Software (ver. 2016a) (Essen Bioscience, Sartorius, Göttingen,) and colony counts were confirmed by manual curation.

### 4.3. Dose Planning and Simulations

The irradiation plan was carried out as a step and shoot intensity-modulated radiotherapy (IMRT) plan, describing the different dose levels as separate target regions. Planning was done with Raystation treatment planning system (RaySearch Laboratories, Stockholm, Sweden) based on a CT scan of the hypoxia chamber containing 96-well plates filled with water. Irradiation was performed on a Siemens Artiste (6 MV) (Siemens, München, Germany).

### 4.4. Modeling Approach 

Large parts of the general model and its derivation were presented and discussed in detail in [11]. Computationally, the code was fully rewritten in Python and elements of GPU computation were introduced. In short, for low LET radiation a homogeneous deposition of dose throughout the cell nucleus with a cell line independent DSB induction rate $\alpha_{DSB}=5\cdot 10^{-3}DSB/\left(Mbp\cdot Gy\right)$, constant over the clinical dose range, wass assumed [36,37,38,39]. Thus, the expected number of DSB in the nucleus ($\langle N_{tDSB}\rangle$) can be expressed as:

(1) $$\langle N_{tDSB}\rangle =\alpha_{DSB}\cdot D\cdot DNA_{c}$$

where DNAc is the DNA content of a cell in Mbp and *D* the applied dose in Gy. The total number of giant loops ($N_{gl}$) with a DNA content of $DNA_{gl}$ inside the nucleus is then given by:

(2) $$N_{gl}=\frac{DNA_{c}}{DNA_{gl}}$$

In this work we assumed DNAc and $DNA_{gl}$ to be 6 Gbp and 2 Mbp, respectively.

We implemented a Monte Carlo routine in which, at each iteration, the number of total DSB in the nucleus ($N_{tDSB}$) was sampled following a Poisson distribution with the expectation value given by Equation (1). After randomly distributing the sampled amount of DSBs over the giant loops, the number of giant loops without any DSB ($N_{0}$), with an isolated DSB ($N_{iDSB}$), or a complex DSB ($N_{cDSB}$) were scored. With the lethality parameters $K_{iDSB}$ and $K_{cDSB}$, which represent the probabilities of an isolated lesion and a complex lesion leading to cell death, respectively, the probability of the cell to survive (*S*) is given by [13]:

(3) $$S={\left(1-K_{iDSB}\right)}^{N_{iDSB}}\cdot {\left(1-K_{cDSB}\right)}^{N_{cDSB}}$$

We obtained the expected fraction of a cell population surviving an irradiation by meaning *S* values obtained from the Monte Carlo algorithm. The lethality parameters can be determined by fitting the result of this routine to survival data.

Experimental evidence suggests that one can assume a homogeneous distribution of oxygen inside the nucleus, no change in DNA content or nucleus volume under reduction of oxygen supply [40], and that the oxygen concentration in the cell does effect the initially induced total number of DSB but not their repair rate [6]. Thus, in our model, a change in oxygenation solely leads to an introduction of a modified DSB induction rate $\alpha_{DSB}^{O_{2}}$, which is given by:

(4) $$\alpha_{DSB}^{O_{2}}=\frac{\alpha_{DSB}}{HRF_{DSB}^{O_{2}}}$$

where $\alpha_{DSB}$ is the rate under normoxia.

The modification of $\alpha_{DSB}$ leads through Equation (1) to a change of $N_{tDSB}$ to $N_{tDSB}^{O_{2}}$, which subsequently leads to alterations of $N_{iDSB}$ and $N_{cDSB}$ to $N_{iDSB}^{O_{2}}$ and $N_{cDSB}^{O_{2}}$, respectively. Keeping the lethality parameters invariant, as implied above, the survival probability of a cell under hypoxic conditions can then finally be written as:

(5) $$S_{O_{2}}={\left(1-K_{iDSB}\right)}^{N_{iDSB}^{O_{2}}}\cdot {\left(1-K_{cDSB}\right)}^{N_{cDSB}^{O_{2}}}$$

The $HRF_{DSB}^{O_{2}}$ value can be determined by fitting the model to hypoxic data, while keeping $K_{iDSB}$ and $K_{cDSB}$ at the values derived from normoxic data. If either one, hypoxic or normoxic data, is not available, the $HRF_{DSB}^{O_{2}}$ can be estimated from the parameterization:

(6) $$HRF_{DSB}^{O_{2}}=\frac{m\cdot K+\left[O_{2}\right]}{K+\left[O_{2}\right]}$$

introduced in our previous work [11], proposed by Carlson et al. [6] and inspired by the initial works of Alper and Howard-Flanders [28]. Fitting this parameterization to data available in literature, we found the values m = 2.94 and K = 0.129% [11].

In order to model the increased cell killing of repair-deficient mutant cell lines or cells exposed to different concentrations of a repair inhibiting drug, a radiosensitization factor (*RSF*) was introduced into the model. The *RSF* modifies the lethality parameter of isolated damages $K_{iDSB}$ only, so that the survival probability of a repair impaired cell reads:

(7) $$S_{-Repair}={\left(1-RSF\cdot K_{iDSB}\right)}^{N_{iDSB}}\cdot {\left(1-K_{cDSB}\right)}^{N_{cDSB}}$$

The RSF is introduced as a free parameter, which is determined by fitting the result of the modified survival probability given in Equation (7) to repair impaired data, while the lethality parameters $K_{iDSB}$ and $K_{cDSB}$ remain set to the values derived from wild type/non-treated cells. As argued above, no interaction between the oxygenation status and the repair capacity is assumed. Therefore, also in the case of a modification of the isolated lesions lethality, it is assumed that the RSF value is set to be invariant under change of the oxygen concentration.

## Figures and Tables

**Table 1:** Model parameters derived from cell survival data of five cell lines irradiated under normoxia and under two hypoxia levels (0.5% and 1% [$O_{2}$]).

Cell Line	$K_{iDSB}$	$K_{cDSB}$	$HRF_{DSB}^{O_{2}}\;1\%\;\left[O_{2}\right]$	$HRF_{DSB}^{O_{2}}\;0.5\%\;\left[O_{2}\right]$
A549	4.83 × 10^−3^ ± 0.88 × 10^−3^	1.69 × 10^−1^ ± 0.31 × 10^−1^	1.60	2.04
H460	3.28 × 10^−3^ ± 1.20 × 10^−3^	2.41 × 10^−1^ ± 0.86 × 10^−1^	1.24	1.44
H1437	3.83 × 10^−3^ ± 0.83 × 10^−3^	1.37 × 10^−1^ ± 0.38 × 10^−1^	1.09	1.33
B16F10	4.05 × 10^−3^ ± 0.44 × 10^−3^	1.34 × 10^−1^ ± 0.18 × 10^−1^	1.29	1.44
Renca	1.67 × 10^−3^ ± 0.18 × 10^−3^	2.04 × 10^−1^ ± 0.06 × 10^−1^	1.28	1.67

**Table 2:** Model parameters derived from cell survival data of CHO wild-type (WT) cells and two DNA-PK response-deficient mutants (V3 and xrs5) irradiated under normoxia and hypoxia (<1% [$O_{2}$]), taken from [30]. HRF, hypoxia reduction factor; RSF, radiosensitization factor.

Cell Line	$K_{iDSB}$	$K_{cDSB}$	$HRF_{DSB}^{O_{2}}$	*RSF* V3	*RSF* Xrs5
CHO WT	4.38 × 10^−3^ ± 1.37 × 10^−3^	2.33 × 10^−1^ ± 0.27 × 10^−1^	2.44	9.60 ± 0.19	14.85 ± 0.50

**Table 3:** Model parameters derived from cell survival data of H1437 and H460 cells irradiated under normoxia and hypoxia (1% [$O_{2}$]) after exposure to different concentrations of an ATM inhibitor.

Cell Line	$K_{iDSB}$	$K_{cDSB}$	$HRF_{DSB}^{O_{2}}$	RSF 100 nM	RSF 200 nM	RSF 500 nM
H460	3.88 × 10^−3^ ± 2.19 × 10^−3^	2.55 × 10^−1^ ± 0.85 × 10^−1^	1.31	1.73 ± 0.15	2.56 ± 0.27	4.21 ± 0.59
H1437	3.11 × 10^−3^ ± 0.86 × 10^−3^	1.50 × 10^−1^ ± 0.35 × 10^−1^	1.10	1.77 ± 0.12	2.52 ± 0.13	3.77 ± 0.15

## Abbreviations

LET	Linear energy transfer
UNIVERSE	Unique and versatile engine
DSB	(DNA) Double strand break
iDSB	Isolated DSB
cDSB	Complex DSB
OER	Oxygen enhancement ratio
HRF	Hypoxia reduction factor
ATM	Ataxia-telangiectasia mutated
NHEJ	Non-homologous end joining
DDR	DNA damage repair
CHO	Chinese hamster ovary
GLOBLE	Giant loop binary lesion
CT	Computed tomography
LQ	Linear quadratic (model)
